# Creatures Great and Small: Horse and Dog Allergy Cross-Reactivity

**DOI:** 10.7759/cureus.52058

**Published:** 2024-01-10

**Authors:** Graey Wolfley, Brittanie Neaves, Christopher Coop

**Affiliations:** 1 Internal Medicine, Joint Base Elmendorf-Richardson Medical Center, Anchorage, USA; 2 Allergy and Immunology, Keesler Medical Center, Biloxi, USA

**Keywords:** military, immunotherapy, dog, horse, cross-reactivity

## Abstract

Cat, dog, and horse aeroallergens can cause allergic disease in susceptible individuals. Allergy cross-reactivity occurs when the body recognizes the protein of one allergen as being similar to a different protein, leading to an allergic response. Cross-reactivity has been demonstrated among animal species such as cat, dog, and horse. These allergens fall into the same protein families, with the lipocalin family predominating. We present a case demonstrating cross-reactivity among cat, dog, and horse allergens. Additionally, our case highlights the difficulty of managing and receiving allergen immunotherapy within the military health system due to the disruption of therapy due to frequent moves and temporary duty assignments.

## Introduction

Allergens from cats and dogs are an important cause of allergic diseases, including allergic rhinitis and asthma [1]. Additionally, horse allergy has been identified in individuals who work around horses [2-3]. Cat, dog, and horse allergens have been found to be cross-reactive [4-6]. Many different allergenic proteins have been discovered when evaluating the cross-reactivity of animals [7]. The aim of this paper is to review the literature on cat, dog, and horse cross-reactivity and to highlight the difficulty of managing allergen immunotherapy (AIT) in the military health system, as members move frequently.

## Case presentation

A 34-year-old, active-duty male orthopedic surgeon with a past medical history of allergic rhinoconjunctivitis was referred to the allergy clinic for evaluation and consideration of AIT. His symptoms consisted of rhinorrhea, sneezing, nasal congestion, and itchy, watery eyes. His symptoms had been present for several years occurring predominantly in the spring and fall but also perennially when exposed to animals such as cats, dogs, and horses. The patient was raised on a ranch where he was exposed to horses and cattle, but his current pet exposures only consisted of a dog.

The patient’s exam was notable for injected conjunctiva, nasal mucosa edema, and a cobblestone throat. His symptoms were not alleviated with oral cetirizine and nasal fluticasone. He had previously been receiving AIT for 18 months, which had improved his symptoms but had not received AIT for six months due to a move to his current duty station.

An aeroallergen skin testing panel was performed with the addition of cattle and horsehair. Skin testing was positive for trees, weeds, molds, cats, dogs, dust mites, and horsehair; cattle hair was negative (Table 1). The risks and benefits of AIT were discussed with the patient who elected to proceed with restarting AIT. He was started on AIT for trees, weeds, molds, cats, dogs, dust mites, and horsehair in addition to counseling on aeroallergen avoidance. He responded well to AIT but, unfortunately, was not able to continue AIT because of a military deployment.

**Table 1:** Allergy skin testing results

	Wheal (mm)	Flare (mm)
Negative Control	0	0
Positive Control	7	8
Environmental		
Cat	10	40
Dog	3	4
Horse	20	24
Cattle	0	0
Mite, Pteronyssinus	7	8
Trees		
American Beech	25	32
Birch	18	19
Box Elder	17	30
Mountain Cedar	27	45
Cottonwood	10	40
American Elm	35	50
Sweet Gum	10	40
Hackberry	5	6
Red Mulberry	4	10
Oak Mix	10	38
Pecan	5	6
Weeds		
Dock and Sorrel	4	5
Wing Scale	3	4
Molds		
Alternaria	25	45
Aspergillus Fumigatus	3	5
Hormodendrum	3	4

## Discussion

Many different allergenic proteins have been discovered when evaluating the cross-reactivity of animals. Specifically, cats, dogs, and horses share some cross-reacting allergens. Albumin has been shown to be an important cross-reactive allergen. Cabañas et al. evaluated 117 cat-sensitized patients for immunoglobulin E (IgE) reactivity using skin-prick tests and radioallergosorbent test inhibitions assays with cat, dog, and horsehair extracts and their purified albumin extracts. They found that 41% of patients sensitized to cats were also sensitized to dogs and horses. Additionally, 21% of these patients had IgE to three albumins. From this study, the authors concluded that albumins from these three animals share some epitopes that account for the cross-reactivity observed in a third of patients sensitized to cat, dog, and horse [5].

Nisson et al. analyzed the sera of 100 patients with a positive IgE response to a dog-dander extract as well as the sera from two patients with an allergy to dogs, cats, and horses who had a positive enzyme-linked immunoassay to Can f 6, Fel d 4, and Equ c 1. These authors discovered that the dog lipocalin allergen Can f 6 is cross-reactive with the cat lipocalin Fel d 4 and the horse lipocalin Equ c 1 [7].

A third study evaluated the sera from patients with allergic symptoms attributable to cat exposure and concomitant allergy to dogs and horses. The authors reported a strong sequence identity between the lipocalin cat allergen Fel d 4 and horse allergen Equ c 1 with the dog allergen Can f 6. Additionally, there was a very high sequence identity of the albumins from dog, cat, and horse allergens. This study concluded that cats, dogs, and horses share many cross-reactive allergens [6].

Finally, an analysis by Hilger et al. of 44 cat- and dog-sensitized patients demonstrated cross-reactivity between cat and dog serum albumins. Additionally, the cat lipocalin Fel d 4 and dog lipocalin Can f 6 were shown to be cross-reactive. The authors also noted that the lipocalins Can f 6 and Fel d 4 showed great homology to the lipocalin Equ c 1, the major horse allergen [8].

In order for patients to receive benefits from AIT, they must build up and maintain their immunotherapy injections for at least three years. The buildup period typically lasts three to six months.

There are several barriers for allergic active-duty military patients to receive AIT. One such barrier is frequent moves. Our patient had allergic rhinoconjunctivitis around cats, dogs, and horses and was found to be sensitized to these extracts. He had received aeroallergen immunotherapy with success but due to a move, he had to restart the buildup phase of AIT.

Another barrier to AIT for active-duty patients is frequent deployments or temporary duty assignments interrupting AIT courses. AIT is not usually given on deployments or temporary duty assignments unless the patient is located near a major military medical center.

To mitigate these issues, the United States Air Force and Army operate allergy extender clinics at smaller bases and overseas locations to facilitate the maintenance of immunotherapy for military patients. These smaller allergy clinics are run by primary care physicians and supervised by regional allergists at major military medical centers via telehealth and electronic/telephonic communication.

Finally, extracts for AIT are not standardized between different manufacturers within the United States or other countries. Fortunately, AIT extracts in the United States military are obtained through the Extract Laboratory Management System (ELMS). Unfortunately, many active-duty patients are evaluated by civilian allergists who do not use ELMS and therefore, when they move, AIT has to be restarted due to the lack of uniformity of the extracts.

## Conclusions

Allergen cross-reactivity is more often recognized in pollens and foods but is also present in animal dander. Our case presents an interesting patient who was allergic to cats, dogs, and horses with cross-reactivity among the allergens. Animal allergen cross-reactivity during the diagnosis and treatment of allergic rhinosinusitis should be considered. Additionally, our case highlights the difficulty of managing AIT in the military health system due to frequent moves, deployments, and temporary duty assignments.
